# Programmatic implications of implementing the relational algebraic capacitated location (RACL) algorithm outcomes on the allocation of laboratory sites, test volumes, platform distribution and space requirements

**DOI:** 10.4102/ajlm.v6i1.545

**Published:** 2017-02-28

**Authors:** Naseem Cassim, Honora Smith, Lindi M. Coetzee, Deborah K. Glencross

**Affiliations:** 1 National Health Laboratory Service (NHLS), National Priority Programmes, Johannesburg, South Africa; 2 Department of Molecular Medicine and Haematology, Faculty of Health Sciences, University of Witwatersrand, Johannesburg, South Africa; 3 Department of Mathematical Sciences, University of Southampton, Southampton, United Kingdom

## Abstract

**Introduction:**

CD4 testing in South Africa is based on an integrated tiered service delivery model that matches testing demand with capacity. The National Health Laboratory Service has predominantly implemented laboratory-based CD4 testing. Coverage gaps, over-/under-capacitation and optimal placement of point-of-care (POC) testing sites need investigation.

**Objectives:**

We assessed the impact of relational algebraic capacitated location (RACL) algorithm outcomes on the allocation of laboratory and POC testing sites.

**Methods:**

The RACL algorithm was developed to allocate laboratories and POC sites to ensure coverage using a set coverage approach for a defined travel time (T). The algorithm was repeated for three scenarios (A: T = 4; B: T = 3; C: T = 2 hours). Drive times for a representative sample of health facility clusters were used to approximate T. Outcomes included allocation of testing sites, Euclidian distances and test volumes. Additional analysis included platform distribution and space requirement assessment. Scenarios were reported as fusion table maps.

**Results:**

Scenario A would offer a fully-centralised approach with 15 CD4 laboratories without any POC testing. A significant increase in volumes would result in a four-fold increase at busier laboratories. CD4 laboratories would increase to 41 in scenario B and 61 in scenario C. POC testing would be offered at two sites in scenario B and 20 sites in scenario C.

**Conclusion:**

The RACL algorithm provides an objective methodology to address coverage gaps through the allocation of CD4 laboratories and POC sites for a given T. The algorithm outcomes need to be assessed in the context of local conditions.

## Introduction

The National Health Laboratory Service (NHLS) of South Africa provides national coordination for the laboratory service, staff training, quality control and quality assurance, as well as managing the overall quality management system. Within the national network of 266 laboratories, CD4 testing is currently offered at 59 laboratories to facilitate the staging and monitoring HIV-infected patients. These laboratories operate all levels of pathology service, including routine diagnostic chemical pathology, haematology and microbiology services. CD4 testing is offered using an integrated tiered service delivery model (ITSDM) that matches daily testing demand with appropriate testing capacity. This is required to manage laboratory workload, turn-around-time (TAT), instrument capacity utilisation and cost.^1,2^ CD4 testing is standardised using Beckman Coulter (Beckman Coulter, Miami, Florida, United States) equipment and the PanLeucoGating platform.^3,4^ In 2014, the NHLS performed 3.9 million CD4 tests.

The ITSDM model aims to ensure efficient, cost-effective provision of quality testing across all health districts.^1^ This ‘full coverage’ model strives toward equitable access to CD4 testing by providing technology that appropriately matches service delivery requirements, based on factors such as test volumes, distances from referring clinics to CD4 laboratories, and the package of clinical services offered by health facilities.^1^

Five testing tiers, along with a sixth coordinating tier, are defined in the ITSDM model: (1) true point-of-care (POC) services (Tier 1), reserved for hard-to-reach areas, where nursing staff attending to patients operate the testing system (< 3 tests per system per day) and initiate patients onto antiretroviral therapy; (2) POC hubs (Tier 2), namely, ‘mini-laboratories’ using only POC equipment for all relevant HIV and tuberculosis tests at a rate of < 10 samples per day in rural health districts; (3) community laboratories (Tier 3) processing less than 100 samples per day; (4) district laboratories (Tier 4) processing between 100 and 299 samples per day; (5) high volume centralised laboratories (Tier 5) processing in excess of 300 samples per day; and (6) Tier 6, representing a national reference/‘monitoring and evaluation’ centre responsible for coordination, harmonisation and standardisation of testing, as well as coordination of training and quality control across a national network of laboratories and related testing sites.^1^

It would also be cost effective to consolidate Tier 4 and/or Tier 5 laboratories into larger centralised laboratories (‘super-laboratory’/Tier 6 level) that could process in excess of 600 samples per day, depending on efficiency of local transport systems.^1^ This would, however, create coverage gaps that would need to be addressed with POC in remote ‘hard to reach’ areas.^1^

An analysis of test volumes identified that the majority of CD4 samples were received from primary healthcare clinics (67%), with a further 8% from larger community healthcare centres; this tells us that hospitals only account for 25% of test volumes.^1^ This confirms the decentralisation of CD4 test requests. Additionally, 14 health districts in South Africa do not have a local in-district CD4 services.^1^ This requires CD4 samples to be referred to distant testing laboratories, which affects both TAT and specimen integrity. As a result, extended TATs were observed for several of these districts, for example, Vhembe and Thabo Mofutsanyane health districts.^1^ By using a relative Euclidian radius of 100 km around each CD4 laboratory (‘service precincts’), both over-and under-subscribed areas were identified.^1^ The under-subscribed areas were predominantly in health districts in the Northern Cape and Eastern Cape provinces,^1^ whereas significant over-subscription of CD4 testing was noted in the KwaZulu-Natal province, specifically in the Ethekwini health district.^1^

The World Health Organization guidelines for a successful health laboratory network state that it is vital to provide equitable access to quality laboratory services, with specific attention focused on rural, semi-urban and underserved areas.^5^

**FIGURE 1:**
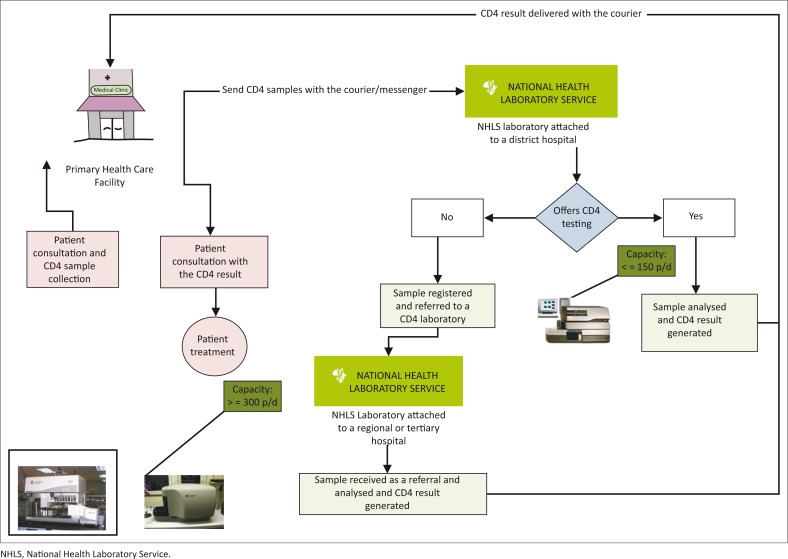
End to end process for CD4 samples from sample collection to using the result during the patient consultation.

The flow diagram (Figure 1) describes this process, detailing all the steps required to deliver a CD4 result to a health facility. CD4 samples are collected by healthcare workers and delivered to the local laboratory by either courier and/or messenger. Where the local laboratory offers CD4 testing, results are delivered following sample analysis. If CD4 testing is not offered at the local laboratory the procedure is as follows: (1) sample registered as a referral on the Laboratory Information System; (2) sample packaged for delivery to the testing laboratory; (3) sample transported by inter-laboratory courier service; (4) samples registered at the CD4 laboratory’s Laboratory Information System; (5) sample tested; (6) result printed at the local laboratory; and (vii) result delivered to the health facility. The NHLS strategic plan (2010–2015) aimed to deliver quality, timely, accessible and customer-focused services.^6^

The current CD4 service has predominantly implemented Tiers 4 and 5 of the ITSDM model. Tier 4 laboratories use the Epics XL MCL^TM^ flow cytometers with the PrepPlus II^TM^ workstation from Beckman Coulter. Tier 5 laboratories use higher throughput platforms with greater automation that could process up to 800 samples per day, depending on the number and configuration of the platforms implemented, for example, the 2 + 1 configuration that consists of two Cellmek^TM^ cell preparation systems and one MPL^TM^ flow cytometer (also from Beckman Coulter). The NHLS is currently replacing the Epics XL MCL^TM^ flow cytometers with the AQUOIS™ load and go system (Beckman Coulter). The different flow cytometer platforms offer daily throughput of up to 386 samples per day and per system, suitable for busy laboratories (Cellmek^TM^ and MPL^TM^). In contrast, the Epics XL MCL^TM^ offers a daily capacity of up to 150 samples per day per system, which is appropriate for smaller laboratories at district hospitals. POC testing was based on the Alere Pima^TM^ platform (Alere Technologies GmbH, Jena, Germany).

A pilot Tier 3 laboratory was implemented at the rural De Aar laboratory^7^ which demonstrated improved access to CD4 testing for the district. The NHLS has a large pool of laboratories currently not performing CD4 testing that could potentially be utilised to rapidly expand services while maintaining quality in a cost-effective approach.^8^

The current challenge the NHLS faces is over-capacitation in urban areas and under-capacitation in selected rural areas. Therefore, the objective of this study was to identify and address coverage gaps, identify over-subscribed areas and assess the impact the relational algebraic capacitated location (RACL) outcomes would have on placement of laboratory sites, test volumes, platform choice and space requirements.

## Methods

### Spatial representation of RACL algorithm output for each scenario

**FIGURE 2:**
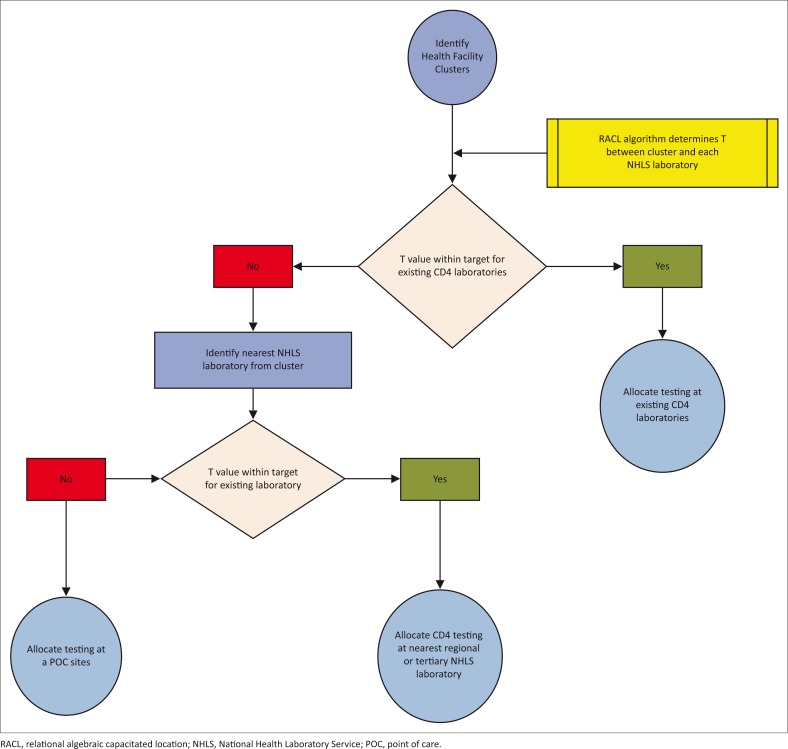
Flow chart of the steps followed by the RACL algorithm.

The RACL algorithm was developed to allocate CD4 laboratories and POC sites to ensure coverage for all health facilities to CD4 testing within a pre-determined travel time (T) using a set coverage approach.^9,10^ Health facilities were clustered within a radius of 5 km to speed up the algorithm; for example, multiple health facilities in the town of Colesberg were clustered due to their proximity. Latitude and longitude data for all health facilities and NHLS laboratories were used to determine Euclidean distances between health facility clusters and NHLS laboratories.^9,10^ Due to financial constraints, Google® Maps Directions API Web Service was used to obtain drive time estimations for a representative sample of Euclidean distances.^9,10^ This dataset was used to generate a linear regression analysis between drive time (hours) and Euclidean distance (km).^9,10^ Based on this analysis, the Euclidean distance dataset for each health facility cluster was converted to travel times.^9^ The algorithm allocates the lowest number of CD4 laboratories and/or POC sites to ensure coverage within the defined T.^9,10^ Health facility clusters outside T are then allocated as POC sites.^9,10^ The logic applied for the RACL algorithm is depicted in Figure 2.

For the purposes of this study, data included 3200 health facilities tested at 62 CD4 testing sites (part of the 266 NHLS laboratories) with a daily capacity of 3600 tests. Provincial and/or NHLS boundaries were not considered for modelling.

The RACL model was repeated for the following travel times:^9,10^

- Scenario A: T = 4 hours
- Scenario B: T = 3 hours
- Scenario C: T = 2 hours.

The algorithm outcomes for the scenarios above were visualised using Google® Fusion Tables as follows:^9,10^

- Red point marker icon (letter A): Existing CD4 laboratories.
- Red point marker icon (letter E): Introduction of CD4 testing at an existing NHLS laboratory (that does not currently offer CD4 testing).
- Small blue circle: POC sites.
- Lines represent the Euclidian distance (km) from the health facility cluster to the CD4 laboratory as follows: (1) green, where T = 2 hours; (2) purple, where T = 3 hours; and (3) orange, where T = 4 hours.

The algorithm outcomes for each scenario included:^9,10^

- Number and placement of CD4 laboratories and POC sites.
- Euclidian distances from the health facility cluster to the allocated CD4 laboratory.
- Daily test volumes for CD4 laboratories.

### Distribution of laboratory test volumes

For each scenario, daily laboratory testing volumes were reported using a line chart. The dataset was sorted from the highest to lowest test volumes, and current daily volumes for 2015 were also reported.

### Analysis of platform and space requirements for the 10 busiest laboratories for each scenario

The top 10 laboratories with the highest daily volumes for each scenario were used to assess the appropriate:

- platform to be deployed, i.e. XL MCL or Cellmek/MPL
- number of platforms required to provide sufficient instrument capacity
- bench space required to accommodate platform(s) based on the supplier width recommendations: for example, the Cellmek requires a bench depth of 1.2 m, which includes the computer, monitor, bottles, cables and tubing.

Additionally, the percentage increase in daily testing volumes from current annual volumes (2015) was assessed.

## Results

### Spatial representation of RACL algorithm output for each scenario

**FIGURE 3:**
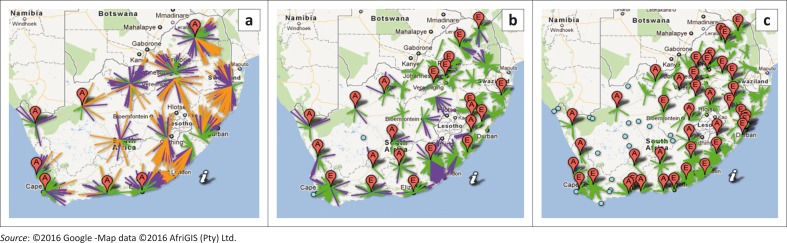
Spatial reporting of (a) Scenario A, (b) Scenario B, and (c) Scenario C using Google® Fusion Tables (copyright of Selective Analytics).

For scenario A, (T = 4), testing would be offered using a fully-centralised approach with only 15 CD4 laboratories (Figure 3 and Table 1). Additionally, CD4 testing would be introduced at the Springbok, Upington and Vredenburg laboratories to meet a T of four hours in the Northern Cape, Western Cape and North West provinces.^9^

**TABLE 1:** Number of CD4 laboratories and point-of-care sites for scenarios A, B and C.

Scenario	T	Laboratories (*n*)	POC sites (*n*)	Total	Current	CD4 laboratory change
A	4	15	0	15	59	-44
B	3	41	2	43	59	-18
C	2	61	20	81	59	2

T, travel time; POC, point of care.

In scenario B (T = 3), the majority of the testing would be performed by Tier 4 (*n* = 24) and Tier 5 (*n* = 6) laboratories with POC reserved for hard-to-reach areas.^1^ Additionally, 11 Tier 3 laboratories were proposed. For this scenario, testing would be retained at 30 of the existing CD4 laboratories. Additionally, CD4 testing would be established at 11 laboratories including Springbok, Beaufort West, Vredendal, De Aar, Upington, George, Graaf-Reinet, Tshwaragano and Thabazimbi laboratories to improve accessibility.^9^

The decentralised scenario C (T = 2) would increase CD4 laboratories to 61, with 20 POC sites.

### Distribution of CD4 laboratories

For scenario A (T = 4), only 15 CD4 testing laboratories are required to ensure equitable access with no POC sites required.^9^ This would imply the closure of 44 CD4 testing laboratories (74.5% reduction in current platform). In scenario B (T = 3), 41 CD4 laboratories were allocated with only two POC sites (CD4 testing will cease at 16 laboratories to be closed). With a halved T value in scenario C (2 hours), 61 CD4 laboratories and 20 POC sites were required to ensure equitable access.^9^ This would require CD4 testing to be stopped at two laboratories and the establishment of 20 new POC sites.

### Distribution of laboratory daily volumes

**FIGURE 4:**
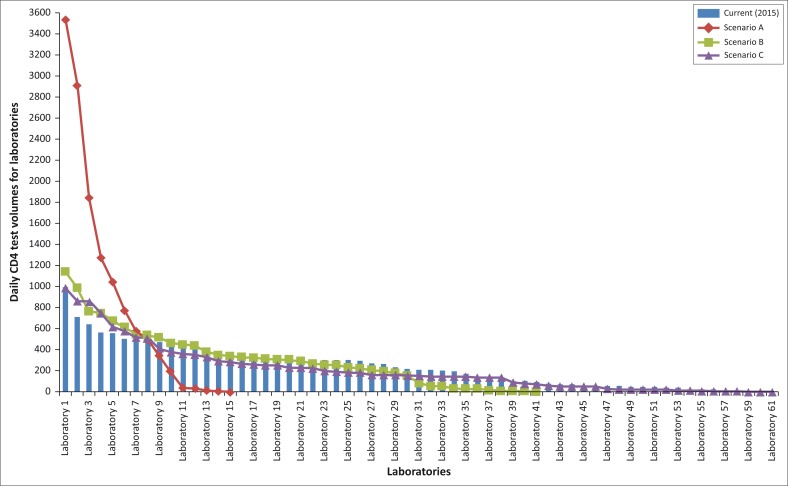
Daily laboratory test volumes for scenarios A, B and C compared to current 2015 volumes.

For scenario A, there would be a significant increase in daily test volumes, with one laboratory required to increase capacity four-fold (Figure 4).^9^ The majority of the CD4 testing would be performed using the Cellmek and MPL platform (nine laboratories would perform between 346 and 3541 tests per day). For high-throughput laboratories, two Cellmek/MPL systems would be able to produce 1080 CD4 results in a 10-hour work day. Even for scenario A, this would require at least four Cellmek/MPL systems to cope with testing demand (capacity of 4320 samples per day).

Daily laboratory test volumes peak at 1142 in scenarios B and 992 in scenario C. These would necessitate two Cellmek/MPL systems. An additional requirement for scenario C is lower-throughput CD4 platforms for laboratories performing between 11 and 50 tests per day (*n =* 8 Tier 3 laboratories).

### Analysis of platform and space impact for the 10 busiest laboratories for each scenario

In scenario A, four laboratories would be required to increase throughput between 87% and 305% (Table 2). At the busiest laboratory, this equates to allocating up to 27 metres of bench space to cope with a daily volume of 3541. To ensure adequate coverage, centralisation is paired with low-volume decentralised laboratories, leaving five laboratories to perform less than 40 samples per day (Tier 2).

**TABLE 2:** Daily laboratory volumes for the 10 busiest laboratories for scenarios A, B and C with the platform, space requirements and percentage increase in volumes compared to 2015 volumes.

Scenario	Daily Vols	Platform required	Qty	Bench width (metres)	% Vols Change
A	3541	Cellmek & MPL 2+2	4	27	255%
	2911	Cellmek & MPL 2+2	3	20	305%
	1849	Cellmek & MPL 2+2	2	14	185%
	1275	Cellmek & MPL 2+2	2	14	124%
	1042	Cellmek & MPL 2+2	1	7	87%
	769	Cellmek & MPL 2+2	1	7	53%
	581	Cellmek & MPL 2+2	1	7	16%
	498	Cellmek & MPL 2+1	1	5	0%
	346	Cellmek & MPL 2+1	1	5	-27%
	194	XL MCL	1	3	-56%
B	1142	Cellmek & MPL 2+2	2	14	14
	992	Cellmek & MPL 2+2	1	7	38
	762	Cellmek & MPL 2+2	1	7	18
	748	Cellmek & MPL 2+2	1	7	31
	670	Cellmek & MPL 2+2	1	7	20
	615	Cellmek & MPL 2+2	1	7	22
	550	Cellmek & MPL 2+1	1	5	10
	538	Cellmek & MPL 2+1	1	5	8
	520	Cellmek & MPL 2+1	1	5	10
	463	Cellmek & MPL 2+1	1	5	5
C	992	Cellmek & MPL 2+2	1	7	-1%
	866	Cellmek & MPL 2+2	1	7	21%
	856	Cellmek & MPL 2+2	1	7	32%
	748	Cellmek & MPL 2+2	1	7	31%
	615	Cellmek & MPL 2+2	1	7	10%
	576	Cellmek & MPL 2+2	1	7	14%
	520	Cellmek & MPL 2+1	1	5	4%
	513	Cellmek & MPL 2+1	1	5	3%
	420	Cellmek & MPL 1+1	1	3	-11%
	379	Cellmek & MPL 1+1	1	3	-14%

For scenario B, the top 10 laboratories are required to make minor changes to their throughput ranging between 5% and 38%. Additionally, the required bench space varies from 5.27 to 13.56 metres. In scenario C, similar increases in throughput are reported at busier laboratories. However, decentralised POC testing reduces volume daily volumes for laboratories 9 and 10.

## Discussion

The RACL algorithm outcomes reported an inverse relationship between T and the numbers of CD4 laboratories/POC sites required to provide coverage. This indicates that the number of CD4 laboratories required to ensure coverage would increase with a decreasing T. As T is increased, it would be possible to ensure coverage with fewer laboratories. The key would be to establish a T that could deliver a clinically-acceptable TAT in line with the standard of care. The current South African guidelines require a CD4 count within seven days to fast-track patients ≤ 200 cells/µL.^11^

The RACL algorithm was repeated for each of three travel times (4, 3 and 2 hours), based on a required TAT of 24 hours. Each scenario provides a solution to address coverage based on a clinically-accepted TAT.

Scenario A represents a highly-centralised model that requires efficient logistics to achieve a T of four hours. The benefits of centralised testing include improved cost efficiency as well as improved testing quality with fewer laboratories.^8^ Challenges include staff recruitment, space availability and infrastructural costs to prepare laboratories for CD4 testing. Additionally, pre-analytical capacity would have to be upgraded to handle increased sample volumes. A key challenge would be higher logistics costs due to increased inter-laboratory referrals that would also affect specimen integrity.

Scenario B offers a mix of predominantly laboratory-based CD4 testing with limited POC testing in ‘hard-to-reach’ areas. Several new CD4 testing sites at existing NHLS laboratories identified have already been implemented by the NHLS. Many of these rural laboratories require limited testing capacity and were therefore identified as Tier 3 laboratories by the ITSDM. A good example is the De Aar laboratory that was implemented in 2012. This laboratory was able to cope with testing demand and reduce TAT substantially from 20.5 to 8.2 hours.^7^ Additionally, with only two staff members, they performed satisfactorily on 10 external quality assessment trials (*n* = 20 samples).^7^ This demonstrates that a small, rural Tier 3 laboratory can integrate CD4 testing for a small incremental cost.^8^

Additional analysis was performed for the National Health Insurance pilot districts (*n* = 11) to assess service gaps.^12^ This study identified that four new tier 3 CD4 testing laboratories would be required to improve access to testing.^12^ These testing sites coincide with the new CD4 testing sites proposed in scenario B.

In scenario C, testing is extended to 61 laboratories with 20 POC sites. Many of the POC sites proposed would be within an acceptable T if a Tier 2 site (POC hub) were established in Calvinia. Furthermore, a Tier 2 site would serve multiple health facilities and offer access to multiple tests (CD4, Xpert MTB/RIF^TM^, creatinine, alanine transaminase and haemoglobin/full blood count).^1^ A Tier 2 site would be more cost-effective than multiple Tier 1 sites.^8^

Across the three scenarios, laboratory-based CD4 testing varies between 15 and 61 sites. For scenario A and B, between 18 to 44 existing CD4 laboratories would have to be consolidated, resulting in significant changes to sample logistics, equipment and staff allocation. Reducing a platform of 62 laboratories to 15 would require a massive scale-up to cope with increased test volumes. This would include a scale-up in the pre-analytical section as well and could incur both renovation and verification costs. Consolidation would require staff to relocate to urban areas; this would need to be handled using a change management approach with additional costs. Additionally, samples would have to travel further, increasing per kilometre logistics costs.

In summary, the RACL algorithm identified the optimal placement of NHLS laboratories for a range of T to enable the delivery of a CD4 service that balances the need for equitable access and cost-effectiveness. However, evidence from the pilot tier 3 laboratory demonstrates that additional tier 3 laboratories or tier 2 POC hubs could increase coverage in a more cost-effective manner than POC sites.

### Limitations

Travel times reported are based on a representative sample of Google® Maps Directions drive times. The drive time for all health facility clusters could not be obtained due to funding availability. Therefore, travel times could be underestimated in areas with poor road infrastructure resulting in additional coverage gaps requiring additional testing sites and/or POC. The RACL algorithm would have to be rerun should any of the assumptions change, namely, TAT, number of health facilities and test volumes. It would be difficult to provide a statistical analysis of the data presented by the RACL algorithm, such as a list of proposed laboratories/POC sites. Additionally, an assessment of local conditions must accompany the findings of the RACL algorithm to ensure optimal coverage.

### Recommendations

Based on the results of this study, it is recommended that the RACL algorithm be implemented on the corporate data warehouse to enable real-time analysis of coverage gaps. The limitations of using Google’s drive times should be addressed. Additionally, the algorithm should be extended to include other priority tests, such as Xpert MTB/RIF^®^, HIV viral load, HIV DNA PCR and pap smear.

### Conclusion

The RACL algorithm provides laboratory management with an objective methodology to identify and address coverage gaps. However, the algorithm outcomes must be assessed in conjunction with local knowledge to address coverage gaps using a sustainable approach.
